# Rostral associations of MRI atrophy of the amygdala and entorhinal cortex across the AD spectrum

**DOI:** 10.1016/j.ynirp.2026.100392

**Published:** 2026-08-10

**Authors:** Michael I. Miller, Yi Xie, Kaitlin M. Stouffer, Can Ceritoglu, Jiabei Li, J. Tilak Ratnanather, Laurent Younes, Arnold Bakker, Nisha Rani, Marilyn S. Albert, Juan C. Troncoso, Meaghan Morris

**Affiliations:** a Department of Biomedical Engineering, Johns Hopkins University, 3400 N Charles St, Baltimore, 21218, MD, USA; b Department of Electrical and Computer Engineering, Johns Hopkins University, 3400 N Charles St, Baltimore, 21218, MD, USA; c The Center for Imaging Science, Johns Hopkins University, 3400 N Charles St, Baltimore, 21218, MD, USA; d Department of Applied Mathematics and Statistics, Johns Hopkins University, 3400 North Charles Street, Baltimore, 21218, MD, USA; e Department of Neurology, Johns Hopkins School of Medicine, 733 N Broadway, Baltimore, 21205, MD, USA; f Department of Psychiatry and Behavioral Sciences and Department of Neurology, Johns Hopkins University School of Medicine, 733 N Broadway, Baltimore, 21205, MD, USA; g Department of Pathology, Johns Hopkins University School of Medicine, 733 N Broadway, Baltimore, 21205, MD, USA; h Kavli Neuroscience Discovery Institute, Johns Hopkins University School of Medicine, 725 N. Wolfe Street, 801 Hunterian, Baltimore, 21205, MD, USA; i Department of Computer Science, Johns Hopkins University, 3400 N Charles St, Baltimore, 21218, MD, USA

**Keywords:** Alzheimer’s disease, MRI biomarkers, Laminar cortical thinning, Amygdala atrophy, Multimodal neuroimaging

## Abstract

This paper examines associations of atrophy in the amygdala, entorhinal cortex and hippocampus based on magnetic resonance imaging (MRI) and Positron Emission Tomography (PET) scans from two independent cohorts: Alzheimer’s Disease Neuroimaging Initiative (ADNI) and Biomarkers of Cognitive Decline Among Normal Individuals (BIOCARD) study. The amygdala, entorhinal cortex (ERC) and transentorhinal cortex (TEC) are shown to change earlier in the disease than the hippocampus based on volume atrophy rate. Over four hundred laminar reconstructions showed that ERC/TEC volume loss is linked to cortical thinning, as a more specific measure historically linked to the layer-specific pattern of tau pathology deposition. Additionally, high-field atlasing with delineations of amygdala subregions shows predominant volume loss in medial subregions including basomedial, basolateral, and corticocentromedial amygdala compared with the lateral subregion. Consistent with prior studies linking MRI-derived atrophy to hyperphosphorylated tau deposition in the amygdala and ERC/TEC, the atrophy rate marker demonstrates strong associations with tau PET measures and spatial co-localization with tau pathology.

## Highlights

• Amygdala and ERC show earlier atrophy than hippocampus in preclinical AD.
• ERC atrophy reflects cortical laminar thinning in surface reconstructions.
• Medial amygdala subregions show greater atrophy than the lateral amygdala.
• MRI atrophy correlates with regional tau PET burden across the medial temporal lobe.

## 1 Introduction

Alzheimer’s disease (AD) remains the leading cause of dementia all over the world. With new clinical therapies and trials targeting the pathology underlying AD, early diagnosis through biomarkers becomes increasingly important. AD neuropathological changes begin many years before clinical symptoms, leading to a new framework for classifying AD pre-symptomatic stages using biomarkers of AD pathology (Jack et al., 2018, Jack et al., 2024). Recent progress in neuroimaging offers opportunity to localize structural and molecular changes in vivo reflecting underlying AD pathologies to improve early detection and diagnostic accuracy for mild cognitive impairment (MCI) and AD dementia.

The medial temporal lobe (MTL) remains especially important in the classic neuropathological staging of early AD. Supratentorial tau pathology first arises in the transentorhinal cortex (TEC) (sometimes considered part of the perirhinal cortex) and adjacent entorhinal cortex (ERC), subsequently extending to involve the hippocampus, before ultimately spreading throughout the neocortex as described by Braak and Braak (Braak and Braak, 1991). This pathological sequence is the basis of the Braak staging of AD tau pathology from I to VI (Braak and Braak, 1991, Braak et al., 2006) and cornerstone of the current system to determine the severity of Alzheimer’s disease neuropathological change (ADNC) (Hyman et al., 2012). The description of this pathological sequence weighs heavily on the early involvement of the ERC followed by hippocampus in stages I or II, but does not focus on the amygdala until stage III (Braak and Braak, 1991, Braak et al., 2006). Following Braak’s landmark observations, histological studies in early clinical stages of AD have targeted predominantly the ERC/TEC, e.g., the loss of neuronal patterns in layer II therein, even in the mildest stages of AD (Gómez-Isla et al., 1996). However, other investigators venturing beyond the ERC/TEC found that other limbic regions such as the amygdala also accumulate pathology relatively early in the disease (Braak and Braak, 1993, Tsuchiya and Kosaka, 1990, Kromer Vogt et al., 1990, Emre et al., 1993, Chu et al., 1997), with tau pathology in the amygdala in AD distributed in specific spatial patterns (Tsuchiya and Kosaka, 1990, Kromer Vogt et al., 1990, Stouffer et al., 2023).

These observations, based on postmortem examinations, are now being complemented by emerging neuroimaging findings of very early atrophy of the amygdala on structural magnetic resonance imaging (MRI) and tau lesions by Positron Emission Tomography (PET) many years prior to the onset of MCI symptoms (Tristão-Pereira et al., 2025, Salman et al., 2024, Murray et al., 2021, Göschel et al., 2023). Likewise, recent tau PET imaging studies show tau pathology in amygdala early in the disease course (Berron et al., 2021, Insel et al., 2020, Leuzy et al., 2022, Lowe et al., 2018, Schultz et al., 2018, Yoon et al., 2022). This early involvement of the amygdala is consistent with its strong interconnections with the entorhinal-hippocampal network and has gained increasing attention in recent research.

Neuronal loss and synaptic injury in AD presumably lead to the measurable atrophy (volume loss) on structural MRI scans that we and others are demonstrating in the ERC and amygdala. In the Alzheimer’s Disease Neuroimaging Initiative (ADNI) cohort, Kulason et al. demonstrated that atrophy of the entorhinal cortex in MRI correlated with clinical severity in AD – patients with smaller ERC volumes in fact tended to have worse memory performance and more advanced symptoms (Kulason et al., 2019). Longitudinal MRI studies further demonstrate that ERC atrophy can precede and predict cognitive decline (Tward and Miller, 2017). Importantly, a changepoint analysis demonstrated that the accelerations in atrophy (i.e., changepoints) occur almost 10 years prior to MCI symptom onset, many years preceding the changepoint for the hippocampus (Younes et al., 2014).

MRI studies targeting the amygdala have been more limited, but evidence indicates the amygdala undergoes early atrophy (Miller et al., 2015). These studies show decreased amygdala volume correlating with decreased memory/cognition (Goerlich et al., 2017, Mori et al., 1997, Mizuno et al., 2000, Horínek et al., 2006, Roh et al., 2011, Poulin et al., 2011) and increased severity of neuropsychiatric symptoms (Wang et al., 2021, Poulin et al., 2011). A recent study by our group using ADNI data showed significantly higher rates of amygdalar atrophy rates for cognitively unimpaired individuals who progressed to MCI than cognitively unimpaired individuals who remained stable (Stouffer et al., 2023).

Recent developments of high-resolution MRI atlases and deep learning segmentation now allow more accurate labeling of MTL substructures. Previously, reliable MTL segmentation has been challenging due to boundary indistinction and tracing variation. In the case of ERC, its proximity to the cerebrospinal fluid in the collateral sulcus and to the optic chiasm can confound automated segmentation algorithms given its thin laminar structure.

Moreover, the use of a Large Deformation Diffeomorphic Metric Mapping (LDDMM) framework enables the computation of thickness atrophy rate in the laminar structures of the ERC far more accurately than before (Ratnanather et al., 2020), allowing us to demonstrate that the volume changes we have seen early in the entorhinal regions are likely associated with laminar cortex thinning. This is important given the apparent loss of neuronal patterns in layer II associated with early disease. Simultaneously, with the advent of our high-field atlases, we have been able to define high-field 11T templates which partition the amygdala into subregions, allowing us to examine the locality the morphometry marker to regions including basomedial amygdala (BMA), basolateral amygdala (BLA), corticocentromedial amygdala (CMA), and lateral amygdala (LA). By employing the high-resolution diffeomorphic mapping of amygdalar subregions, we can explore the sub-nuclear atrophy patterns within cohorts – seeking to determine if the selective vulnerability of the medial amygdala observed is recapitulated in our sample.

This paper examines data from two independent cohorts, the Alzheimer’s Disease Neuroimaging Initiative (ADNI) study and the Biomarkers of Cognitive Decline Among Normal Individuals (BIOCARD) study, which include data from cognitively unimpaired individuals, those with MCI and those with mild AD dementia. The questions that we examine are four-fold: (1) Do the ERC and amygdala in both samples demonstrate greater atrophy than the hippocampus early in the course of AD, (2) Is the atrophy of ERC volume linked to cortical thinning, as maybe implied by the loss of layer II neurons in early disease, (3) Given progress in high-field imaging and sub-parcellations, can the earliest morphometric changes occurring in the amygdala be tied to its local neighborhood geometry and presumed circuitry with the entorhinal cortex, and (4) Given the stability of the MRI-based atrophy marker, and prior evidence by our group that tau deposition in brain tissue is associated with MTL atrophy (Stouffer et al., 2023), can we demonstrate an association between the MRI atrophy markers and tau burden, as measured by PET. These efforts are intended to advance the development of sensitive and specific imaging biomarkers for the preclinical and prodromal stages of AD, when therapeutic interventions may be most effective.

## 2 Material and methods

Data were obtained from the BIOCARD study and the ADNI database. The BIOCARD Study (www.biocard-se.org) was established in 1995. It was originally led by investigators at the National Institutes of Health (NIH) and was transferred to the research team at the Johns Hopkins School of Medicine in 2009. It is supported by funding from the National Institute on Aging and is led by Dr. Marilyn Albert. The overarching goal of the study is to improve understanding of the earliest phases of AD, accelerate the development of new treatments that are directed at the basic mechanisms of the disease and hasten the time when effective treatments for AD and related disorders become a reality. All procedures were performed in compliance with relevant laws and institutional guidelines and were approved by the appropriate institutional review board/ethics committee. The Johns Hopkins Medicine IRB protocol number for this study is NA_00027232; the most recent approval date was April 21, 2026.

ADNI (https://adni.loni.usc.edu) was launched in 2003 as a public–private partnership, led by Principal Investigator Michael W. Weiner, MD. The primary goal of ADNI has been to test whether serial MRI, PET, other biological markers, and clinical and neuropsychological assessment can be combined to measure the progression of mild cognitive impairment (MCI) and early AD. As a longitudinal multi-center study, ADNI was conducted across successive phases, each operating under its own IRB-approved protocol at participating institutions. All ADNI participants provided written informed consent prior to enrollment. Relevant clinical protocols, protocol versions, and implementation dates are publicly available through the official ADNI Documentation website (https://adni.loni.usc.edu/help-faqs/adni-documentation/).

Details regarding data security, anonymization, and access protocols for both datasets are provided in Appendix A, Appendix B.

### 2.1 BIOCARD demographics and imaging data

Participants in BIOCARD study were categorized into four diagnostic groups based on longitudinal clinical evaluations: cognitively normal (CN), impaired not MCI, MCI, and AD. Subjects in all groups were required to have at least three 3T MR scans for longitudinal comparison. Demographic summaries, including baseline age, scan count, and follow-up duration, are provided in Table 1.

All MRI data were acquired on a 3T MRI system using a 32-channel head coil (Achieva, Philips Healthcare, Best, The Netherlands). A 3D T1-weighted Magnetization-Prepared-Rapid Gradient-Echo (MPRAGE) image was acquired with the following parameters: TR = 6.5 ms, TI = 843 ms, TE = 3.11 ms, turbo factor = 240, shot interval = 3000 ms, flip angle = 8°, voxel size = 1 × 1 × 1 mm${}^{3}$, FOV = 240 × 256 × 204 mm${}^{3}$, and scan duration = 5 min 59 s. Longitudinal 1.5 T structural MRI data acquired with both sagittal and coronal slice orientations were additionally used to validate the robustness of the estimated atrophy-rate patterns. Sagittal acquisitions had a voxel resolution of 1.5×0.9375×0.9375 mm${}^{3}$, while coronal acquisitions had a voxel resolution of 0.9375×2.0×0.9375 mm${}^{3}$. Detailed acquisition parameters for these scans have been previously reported (Younes et al., 2014).

PET scans were acquired on a GE DISCOVERY RX PET/CT scanner in three-dimensional acquisition mode. For tau imaging, ^18^F-MK6240 PET was performed 90 ± 1 min after intravenous injection of 5.0±10% mCi of tracer, followed by a saline flush, yielding six 5-min frames. For amyloid-β imaging, ^11^C-Pittsburgh compound-B (^11^C-PiB) PET was obtained 40 min post-injection of 15 ± 1.5 mCi, reconstructed into four 5-min frames. Residual syringe activity was measured to ensure accurate dose quantification. Image reconstruction used the 3D ordinary Poisson ordered-subset expectation–maximization (3D OP-OSEM) algorithm with corrections for detector efficiency, decay, dead time, attenuation, and scatter (Hudson and Larkin, 1994). Attenuation correction employed low-dose CT (120 keV, 80 mA, slice thickness = 3.75 mm, separation = 3.3 mm, FOV = 500 mm) reconstructed with the standard kernel. Final PET volumes consisted of 2×2×3.27 mm${}^{3}$ voxels representing radioactivity concentration (Bq/mL).

**Table 1:** BIOCARD demographics for four subgroups of normal, impaired not MCI, MCI, and Dementia. Statistics reported as mean ± standard deviation.

Dataset	BIOCARD (n = 130)			
Cognitive group	Control		MCI/AD	
	Normal	Impaired not MCI	MCI	Dementia
# Subjects	51	40	31	8
Baseline age	67.59 ± 6.74	68.68 ± 6.08	70.29 ± 7.86	70.63 ± 6.54
# Scans	3.73 ± 0.60	3.80 ± 0.78	3.68 ± 0.69	3.38 ± 0.48
Scan range	6.67 ± 1.29	6.90 ± 1.55	6.55 ± 1.60	5.75 ± 1.64

### 2.2 ADNI demographics and imaging data

Table 2 depicts the diagnostic groups of the included participants in the ADNI datasets grouped into Controls, which contain CN and SMC (Subjective Memory Concern), MCI which consists of EMCI (Early Mild Cognitive Impairment), MCI and LMCI (Late Mild Cognitive Impairment), and AD. Subjects were required to have at least three 3T MR scans for longitudinal volume comparison.

T1-weighted MRI images were acquired across multiple sites on GE, Philips, and Siemens scanners at 3.0 T. Sequences typically included 3D MPRAGE and 3D Inversion Recovery Spoiled Gradient Recalled Echo (IR-FSPGR). Acquisition parameters varied across protocols, with repetition times (TR) typically 2300 ms (Gradient TR 6.8 ms for Philips), echo times (TE) from 2.8 to 4.1 ms, inversion times (TI) 400/900/1000 ms when applicable, and flip angles $8^{\circ }\text{–}11{}^{\circ}$. Imaging was performed with voxel dimensions of 1.2×1×1 mm^3^, fields of view (FOV) 240/256×256×176–211 mm^3^. All acquisitions employed multi-channel head coils (8–32 channels depending on scanner model).

PET imaging was conducted using ADNI-qualified PET/CT scanners including GE, Philips, and Siemens systems in three-dimensional acquisition mode. Amyloid-β PET imaging was conducted with [^18^F]AV-45 (Florbetapir), administrated as 370±10% (10.0 ± 1.0 mCi) MBq intravenous bolus, with imaging beginning 50 min post-injection and reconstructed into four 5-minute frame. Tau PET imaging was conducted with [^18^F]AV-1451 (Flortaucipir), administered as a 370±10% (10.0 ± 1.0 mCi) MBq intravenous bolus, with imaging beginning 75 min post-injection, reconstructed into six 5-minute frames. All radiotracer injections were followed by a 0.9% sterile sodium chloride flush, and residual syringe activity was recorded for accurate quantification of injected dose. Attenuation correction was performed using low-dose CT scans, typically four 5-min frames for Florbetapir and Florbetaben, and six 5-min frames for AV-1451. Reconstruction parameters vary among different scanners, typically using OSEM-based algorithms (Hudson and Larkin, 1994). Final PET voxel values represent the scanner-reconstructed activity concentration, typically proportional to radioactivity concentration (Bq/mL).

**Table 2:** ADNI demographics for subgroups of Control (CN + SMC, 387 subjects), MCI (EMCI + MCI + LMCI, 523 subjects) and AD (109 subjects). Statistics reported as mean ± standard deviation.

Dataset	ADNI (n = 1019)			
Control	CN		SMC	
# Subjects	346		41	
Baseline age	73.15 ± 6.36		72.39 ± 5.27	
# Scans	6.72 ± 3.93		5.68 ± 2.53	
Scan range	5.69 ± 3.45		4.01 ± 2.28	

MCI/AD	EMCI	MCI	LMCI	AD

# Subjects	210	188	125	109
Baseline age	71.44 ± 7.06	72.88 ± 7.80	72.38 ± 8.16	74.40 ± 7.74
# Scans	9.54 ± 3.92	6.01 ± 4.06	7.75 ± 3.47	5.14 ± 2.30
Scan range	3.95 ± 2.70	4.87 ± 3.43	2.95 ± 2.16	1.52 ± 0.91

### 2.3 Preprocessing of MRI and Amyloid and Tau PET data

All T1-weighted structural MRI scans and PET datasets were processed using a standardized pipeline. For each subject, raw DICOM directories were scanned to identify all available PET acquisitions and classify them as either amyloid or tau. T1-weighted MPRAGE MRI scans were likewise identified and converted from DICOM to NIfTI format using the dcm2niix conversion software (Li et al., 2016). PET series were converted to NIfTI format according to their acquisition protocol, resulting in either 4 ×  5-minute or 6 ×  5-minute dynamic 4D volumes.

Dynamic PET data were preprocessed using SPM12 (Penny et al., 2011). All temporal frames were realigned to the first frame to correct for intra-scan motion, and a static PET volume was generated by simple unweighted averaging of the motion-corrected frames. Each mean PET volume was paired with the temporally closest T1-weighted MPRAGE MRI based on acquisition dates. Rigid-body PET-to-MRI coregistration was performed using FSL FLIRT (Jenkinson and Smith, 2001). Registration quality was verified through visual inspection of PET–MRI overlays in all three orthogonal planes, and datasets showing inadequate alignment were excluded or reprocessed using FSL tools in place of SPM when necessary.

T1-weighted MPRAGE images were additionally processed for skull stripping using DeepBrain, an open-source TensorFlow-based brain-extraction toolkit (Itzcovich, 2025). DeepBrain applies a convolutional neural network to generate a brain-only mask that removes scalp, skull, and other non-brain tissues. All brain-extracted images were visually inspected to ensure accurate preservation of cortical and subcortical anatomy prior to subsequent segmentation.

### 2.4 Regional segmentation

Manual segmentations of medial temporal lobe (MTL) subregions were produced on clinical 3T scans and ex vivo 11.7T scans by a team of two individuals, guided by a neuroanatomist (Stouffer et al., 2023). Segmentations were performed using Seg3D (Scientific Computing and Imaging Institute (SCI), 2016), primarily in the coronal plane. Operational choices to improve boundary precision and consistency included contrast and brightness adjustment before tracing, the use of a 1-voxel brush for fine delineation, and cross-referencing with axial and sagittal views. For all 3T MRI scans, ERC and TEC boundaries follow the protocol used previously and were tied to established neuroanatomical descriptions (Stouffer et al., 2023, Kulason et al., 2019, Kulason et al., 2020, Tward and Miller, 2017, Tward et al., 2017). The anterior boundary of the combined ERC/TEC was defined as 4 mm anterior to the hippocampal head, and the posterior boundary as 2 mm posterior to the gyrus intralimbicus (GI) (Insausti et al., 1998). The lateral boundary of the ERC and the medial boundary of the TEC were defined at the medial bank of the collateral sulcus (CoS), while the lateral boundary of the TEC extended to the deepest point of the CoS. The amygdala was manually segmented in coronal slices, following a protocol based on intensity contrast and comparison with anatomical landmarks identified for the hippocampus (Stouffer et al., 2023). The posterior boundary was first identified between the amygdala and hippocampus using the dark contrast of the inferior horn of the lateral ventricle above the hippocampus. The amygdala was then distinguished by an intermediate gray intensity level (as opposed to posterior white matter) with relative uniform contrast and typically an increasing coronal area with each successively anterior slice. Its anterior boundary was defined by the disappearance of the contrast, occurring on average 11.8 ± 2.0 mm from the posterior border. For consistency, a one-voxel unlabeled gap was intentionally left between the amygdala and the ERC/TEC. In the ADNI dataset, 19 scans were manually segmented in the right hemisphere, while the remaining scans were segmented in the left hemisphere. All BIOCARD scans were manually segmented in the right hemisphere.

MRI scans were all segmented individually to maximize the accuracy of each segmentation. A workflow decision was used to reduce inter-rater variance, where one rater only segmented amygdalae, while the other segmented ERC+TECs. Both raters underwent one month of training in which they practiced on similar structures across different 3T MRI datasets before segmenting the selected ADNI subset. For reproducibility, the study reports intra-rater variability by replicate segmentations of the same structure on the same scan by the same individual at different times, using Dice overlap. Average overlap between two replicated segmentations was 0.87 ± 0.04, with 4−11 days between segmentation attempts (Stouffer et al., 2023).

For ex vivo 11.7T MRI, MTL regions included amygdala, ERC, CA fields, subiculum, presubiculum, parasubiculum, and dentate gyrus. Region boundaries were defined using intensity differences and published MR segmentations (Berron et al., 2017, Wisse et al., 2012, Yushkevich et al., 2009). The amygdala was further subdivided into five individual regions based on previously published delineations (Amunts et al., 2005, Saygin et al., 2017, Kedo et al., 2018), including basolateral (BLA), basomedial (BMA), combined cortical, medial, and central (CMA), and lateral (LA) nuclei and periamygdaloid area (PA). Briefly, subregions were delineated on coronal slices posterior to anterior, with the posterior boundary of the amygdala comprised of CMA, and moving anterolaterally, the posterior boundaries of BMA, BLA, and LA respectively defined. PA was differentiated specifically from the cortical region of CMA by the presence of laminated grey levels. LA typically extended to the anterior most part of the amygdala, with dark contrast lines likely representing amygdalafugal pathways seen in the anterior dorsal region.

### 2.5 nnU-Net MTL segmentation, training data, atrophy rate calculations

**Fig. 1:**
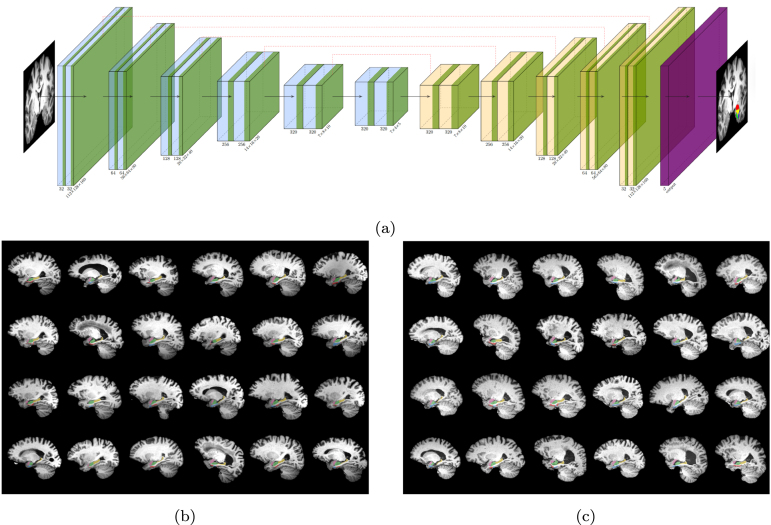
(a) 3D nnU-Net architecture with an encoder–decoder structure. Each level consists of convolutional blocks (Conv–InstanceNorm–LeakyReLU), with downsampling via strided convolutions and upsampling via transposed convolutions. (b) and (c): Sagittal sections of training T1 MRI with manual segmentation in (b) ADNI, and (c) BIOCARD. T1 scans overlaid with segmentation at 40% opacity, including amygdala (pink), ERC (blue), TEC (red), hippocampus anterior (green) and hippocampus posterior (yellow).

Manual segmentations of the 3T MRI brains were used to develop the nnUNet model for the five MTL structures: amygdala, entorhinal cortex (ERC), trans-entorhinal cortex (TEC), hippocampus anterior, and hippocampus posterior. Hippocampus segmentations were generated using ASHS (Yushkevich et al., 2015), edited manually and combined with the aforementioned manual delineation of amygdala and ERC/TEC. Table 3 depicts the statistics of the training data including the 294 ADNI scans and 371 BIOCARD manual segmentations. A collage of the training data with manual segmentations superimposed over the MRIs are shown in Figs. 1(b) and 1(c).

The architecture of the nnU-Net (‘no new net’) (Isensee et al., 2021) we trained for the segmentation protocol is depicted in Fig. 1(a). nnU-Net is a self-configuring image segmentation framework that automatically adapts components of a deep learning pipeline without manual tuning. It analyzes dataset properties such as image size, voxel spacing, intensity properties, etc. to determine preprocessing steps for resampling, normalization, cropping as well as network architecture including 2D/3D U-Net variants, depth, patch size, batch size, and training strategies including data augmentation, loss functions, and learning rate schedule. It then trains multiple configurations (e.g., 2D, 3D full-resolution) and selects the best-performing models. For the MTL segmentations, the 3D full-resolution configuration used a 112 × 128 × 160 patch size with batch size = 2 at 1.2×1×1 mm${}^{3}$. The manual segmentation was partitioned into training and testing subsets using a 90:10 split. The training objective incorporated focal loss, cross-entropy loss, and Dice loss into a unified loss function for optimization. To enable dual-hemisphere prediction, flip augmentation was disabled during training, and all manual segmentations were standardized to a single hemisphere (left hemisphere for ADNI and right hemisphere for BIOCARD). During inference, both the original and left–right flipped images were processed by the model. The resulting segmentations were then flipped back and fused to obtain a dual-hemisphere medial temporal lobe (MTL) segmentation. The held-out test set was used exclusively for final performance assessment with baseline methods, including MRICloud (Mori et al., 2016), FreeSurfer (Fischl et al., 2002), and ASHS.

Atrophy rates were calculated for each subject using longitudinal volume estimates derived from region-specific segmentation masks. Volumes were computed by multiplying the number of voxels within each label by the voxel dimensions (1.0×1.0×1.2 mm${}^{3}$). A linear regression model was fitted to each subject’s longitudinal volume trajectory to estimate the annualized rate of change, expressed as percentage volume change from baseline (%/yr). Standard deviation of all predicted segmentations in each region (amygdala, ERC, TEC, anterior hippocampus, and posterior hippocampus) are calculated, and time points with regional volumes exceeding 2.5 standard deviations from the subject-specific mean were labeled as outliers and excluded from subsequent analysis. Visual inspection was also performed to remove outliers due to poor image quality.

**Table 3:** Statistics of manual segmented training data for ADNI and BIOCARD; nnU-Net was trained separately on each dataset.

	ADNI	BIOCARD
# Scans	294	371
# Subjects	67	107
# of time points per subject	4.39	3.47

### 2.6 LDDMM-based registration and amygdala subnuclei analysis

**Fig. 2:**
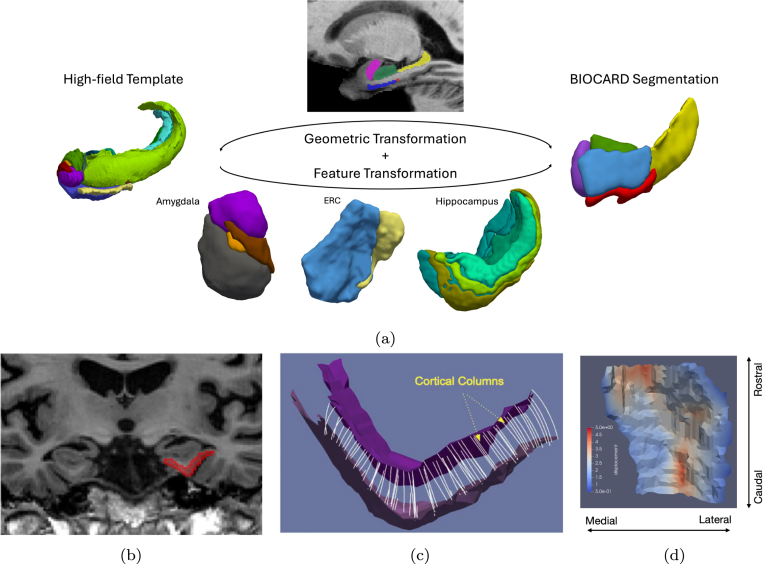
(a) Diffeomorphic mapping from high-field template to BIOCARD and ADNI segmentations depicting both geometric transformation and transfer of template labels. (b), (c) and (d) show reconstruction of ERC/TEC thickness profiles: delineation of ERC/TEC (red) in MRI section (b); LDDMM shooting of cortical columns (c); calculation of thickness scalar field (d).

Fig. 2 depicts the LDDMM frameworks used for projecting the high-field MTL atlases into 3T MRI volumes, as well as the methodology for computationally reconstructing the cortical columns forming the ERC/TEC. Fig. 2(a) illustrates transformations that generate geodesic flows of diffeomorphisms, thereby preserving the topological structure of the anatomy (Beg et al., 2005). Let $I_{0}$ and $I_{1}$ $:R^{3}\to R$ denote the source and target images, respectively. LDDMM seeks a time-dependent velocity field v(x,t) on a suitable smoothness space V (a reproducing kernel Hilbert space, RKHS) that minimizes a variational energy:

(1) $$E\left(v\right)=\int_{0}^{1}∥v_{t}{∥}_{V}^{2}dt+D_{varifold}\left(I_{0}\circ \varphi_{1}^{-1},I_{1}\right),$$

where $\varphi_{1}=\varphi_{0}+\int_{0}^{1}v_{t}\left(\varphi_{t}\right)dt$, with $\varphi_{0}\left(x\right)=x$ is the resulting diffeomorphic warp at t=1; $\int_{0}^{1}∥v_{t}{∥}_{V}^{2}dt=\int_{0}^{1}\left(\sum_{i,j}v_{i}^{T}K\left(x_{i},x_{j}\right)v_{j}\right)dt$ is a smoothness regularizer using RKHS; $D_{varifold}\left(\cdot ,\cdot \right)$ is a data fidelity between deformed source $I_{0}\circ \varphi_{1}^{-1}$ and $I_{1}$ via a varifold-based shape dissimilarity metric that compares the distribution of labeled surface/region elements. The optimal solution of this variational problem corresponds to a geodesic path in the diffeomorphism group connecting $I_{0}$ to $I_{1}$. LDDMM is used to project segmented MTL structures from the high-field atlas (Stouffer et al., 2023) onto 3T MRI targets in ADNI and BIOCARD datasets.

To calculate the atrophy rates within the amygdala subregions we mapped the high-field template to each subject’s 3T segmentation. MTL segmentations across all time points were jointly aligned using varifold LDDMM (Miller et al., 2024). A subject-specific midpoint template was then generated by averaging initial momenta and deforming the high-field atlas. The resulting template was partitioned into amygdala, combined ERC/TEC, and hippocampus, and each structure was mapped to the individual time points (Beg et al., 2005).

Voxel-wise segmentation masks were reconstructed by interpolating the anatomical boundaries onto a regular grid and assigning each voxel to the subregion with the highest empirical likelihood. Atrophy rates of different subregions of amygdala in different disease groups were then computed using methods described in Section 2.5.

### 2.7 Entorhinal cortex reconstruction and cortical thickness calculation

To quantify cortical thickness within the ERC and TEC, we employed a surface-based diffeomorphic framework inspired by prior work in longitudinal shape analysis of the medial temporal lobe. We treated the ERC/TEC together as a single structure to assess thickness continuously across the medial-lateral and anterior-posterior extent. Figs. 2(b)–2(d) depict the pipeline for cortical thickness mapping (Ratnanather et al., 2020). We estimated vertex-wise cortical thickness by computing the geodesic flow between paired cortical surfaces: the inner (white matter) and outer (pial) boundaries. This was done by applying LDDMM to register the inner surface to the outer surface with the additional constraint that the deformation is restricted to the surface normal direction. We used the lengths of the flow lines, provided by each vertex trajectory under the transformation, to define the vertex-wise thickness between the cortical boundaries. The median of the distribution of the vertex-wise thickness was used to attribute a scalar thickness measurement to each ERC/TEC volume. Fig. 2(d) shows the thickness color scale across the ERC/TEC surface.

Thickness atrophy rates per subject was measured in the scalar fields representing the ERC/TEC laminar extent as percentile change in thickness per year (%/year). Among all the generated surfaces, the TEC partitions that were excessively thin were flagged and were removed from the analyses and not included in the calculation of volume-thickness atrophy correspondence.

Finally, we used the LDDMM triangulated mesh framework with hybrid elastic energy (Vaillant and Glaunès, 2005, Younes, 2018, Charon and Younes, 2023) to transport the thickness scalar field on the target surfaces onto the common coordinate system of a template estimated averaging all baseline outer surfaces (Ma et al., 2010).

### 2.8 PET accumulation of tau and amyloid tangles

**Fig. 3:**
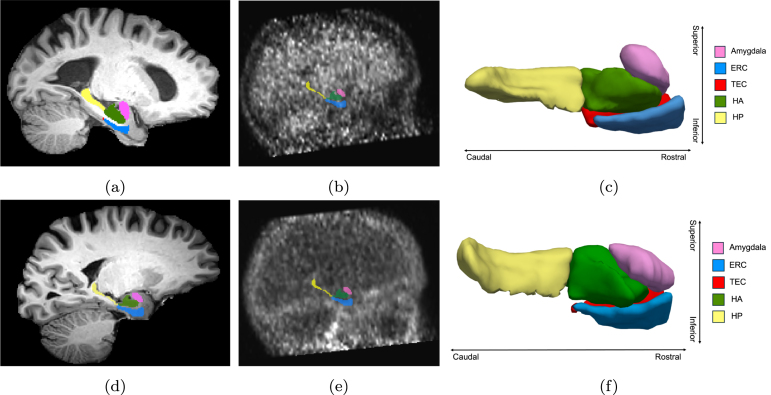
Sagittal sections of T1-weighted image for a subject in (a) ADNI and (d) BIOCARD with (b) tau and (e) amyloid PET images. 3D segmentation of a subject in ADNI (c) and BIOCARD (f) dataset.

Figs. 3(b) and 3(e) respectively show typical tau and amyloid images. For region-specific PET characteristics, we computed the Standardized Uptake Value Ratio (SUVR) for each region of interest (ROI) (Vemuri et al., 2017). For each MTL ROI, the radioactivity concentration (Bg/mL) was calculated by dividing the sum of voxel intensity by its volume. The intensity ratio within an ROI was then defined by normalizing this value by the reference tracer concentration averaged across cerebellar gray matter voxels:

(2) $$\text{SUVR}=\frac{\underset{\left(i,j\right)\in M_{\text{ROI}}}{\sum }I\left(i,j\right)/V_{\text{ROI}}}{\underset{\left(i,j\right)\in M_{\text{Cerebellum GM}}}{\sum }I\left(i,j\right)/V_{\text{Cerebellum GM}}},$$

where I(i,j) denotes the PET intensity at voxel (i,j), M denotes voxel ensembles of different regions, $V_{\text{ROI}}$ and $V_{\text{cerebellum GM}}$ represent the volumes of the ROI and cerebellum grey matter, respectively. Cerebellar gray matter segmentation was generated using MRICloud, an atlas-based automated brain segmentation platform (Mori et al., 2016). The resulting SUVR provides a comparative measure of the signal intensity within a given anatomical structure relative to the non-MTL brain tissue.

### 2.9 Diagnostic classification beyond hippocampal measures

To evaluate whether non-hippocampus MTL structures provide diagnostic information beyond hippocampus measures in early AD, we performed a quantitative classification between CN (346 subjects) and EMCI (210 subjects) using subject-level volumetric and longitudinal atrophy features derived from the amygdala, ERC/TEC and hippocampus. First, we generated a unified longitudinal table containing all eligible ADNI subjects, regional volumes, volume atrophy rates, and demographic information, including sex, diagnosis, acquisition date, and age at scan. Volumes and volume atrophy rates were computed from the nnUNet-predicted bilateral segmentations using the pipeline described in Section 2.5.

We then evaluated whether amygdala and ERC/TEC provided additional diagnostic information beyond hippocampal measures. Specifically, the feature set including amygdala and ERC/TEC measures was compared with the feature set with only hippocampal measures. Each set incorporated average regional volume, annualized volume atrophy rate, volume–atrophy interaction terms, and, where applicable, volume and atrophy ratios relative to the hippocampus. Average age was included as a covariate. These features were selected to capture both absolute structural degeneration and regional vulnerability relative to the hippocampus. A binary classification analysis was conducted to differentiate CN and EMCI subjects. For each feature set, we performed ten repeated 80/20 train-test splits with stratification based on diagnosis, sex, and age tertiles to maintain balanced distributions across training and testing sets. Features were standardized using z-score normalization, and logistic regression models were trained with a maximum of 1000 iterations and regularization parameter C=1. Classification performance was evaluated using the area under the ROC curve (AUC), accuracy, sensitivity, and specificity. For each repeated train-test split, the difference in false-positive rate (FPR) between the hippocampus-only and amygdala+ERC/TEC models was calculated such that positive values indicate improved specificity of the amygdala+ERC/TEC model. Pairwise comparisons between feature sets were performed using Wilcoxon signed-rank tests on the AUC measurements.

## 3 Results

### 3.1 MRI segmentations of BIOCARD and ADNI

Data from the 130 BIOCARD study participants and 1019 ADNI participants were available for analysis. Table 1, Table 2 provide details about the number of participants, diagnostic categories at follow-up, the number of MRI scans included in the analyses and average years of follow-up.

Representative examples of structural MRI imaging from participants in the BIOCARD and ADNI cohorts are shown in Fig. 3. T1-weighted sagittal slices illustrate anatomical integrity and contrast in both datasets, capturing regional patterns of AD-related pathology. Structural consistency across cohorts supports joint analysis of volumetric trajectories. Figs. 3(c) and 3(f) show 3D renderings of the segmentations predicted from the T1-weighted images using our pipeline. Surface meshes were reconstructed and smoothed using the ITK-SNAP toolkit (Yushkevich et al., 2006). Visualizations from both datasets demonstrate the segmentation algorithm’s ability to generate anatomically plausible labels in regions such as the amygdala, ERC, TEC, and hippocampus, serving as the basis for quantitative morphometry analyses.

### 3.2 Deep learning segmentations are consistent and accurate relative to manual raters and other methodologies

**Fig. 4:**
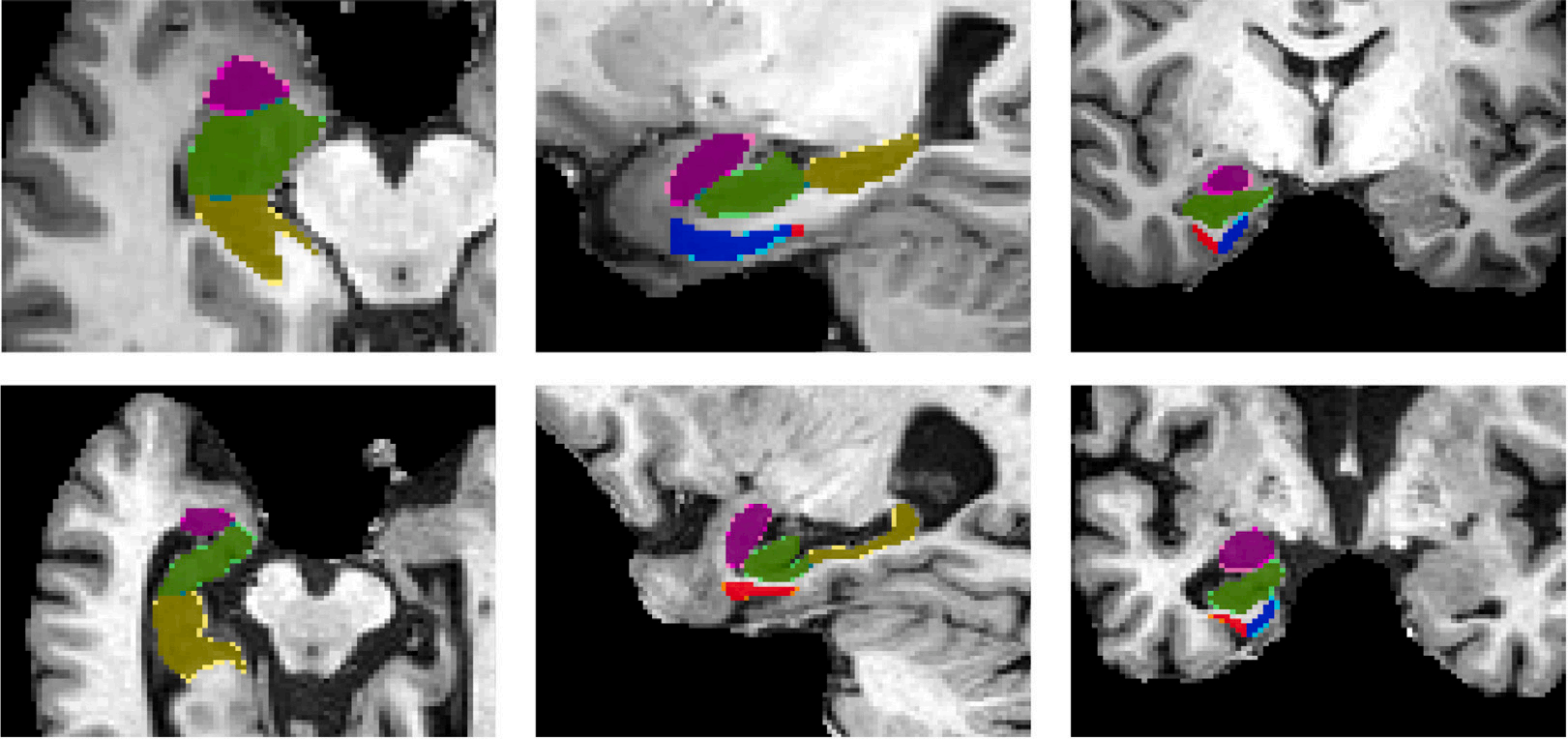
Overlap of manual and nnU-Net predicted segmentation in BIOCARD (top) and ADNI (bottom); dark colors denote overlaps and light denote differences.

Fig. 4 shows overlaps of manual and nnU-Net-predicted segmentation in BIOCARD (top) and ADNI (bottom) in the test datasets. The dark colors denote overlapping voxels, while light colors denote differences between manual and predicted segmentations. Segmentation accuracy was quantitatively evaluated on held-out test subsets using Dice similarity coefficients, comparing nnU-Net predictions to manual segmentations. As visualized and summarized in Table 4, the classifier trained on hundreds of hand segmented MTLs exhibits high Dice scores across all five regions of interest (ROIs) in both the BIOCARD and ADNI datasets, with those within the ERC and TEC similar to those reported for intra-rater reliability of manual segmentations in this area (0.85-0.88) (Sadeghpour et al., 2025). Notably, segmentations of anterior and posterior hippocampus achieved Dice scores exceeding 0.95 in BIOCARD and above 0.92 in ADNI. The amygdala, ERC, and TEC exhibited slightly lower Dice scores, particularly in ADNI, where image contrast varied more substantially across subjects. This reduction also reflects the geometric and cytoarchitectonic complexity of the ERC and TEC regions as laminar structures, as well as the reported variability across human architecture in these areas (Sadeghpour et al., 2025), which pose greater challenges for automated segmentation. Compared to other methods such as Freesurfer, MRICloud and ASHS, nnU-Net achieves high dice similarity coefficients with fast inference, which can be attributed to its training on MTL-specific segmentation that bias the model toward high accuracy within this anatomical region. A detailed comparison of performance across different segmentation tools is given in Appendix C.

We also compared the nnU-Net atrophy rates to other methods. We found that the atrophy rates obtained from the nnU-Net applied to the entire MTL gave similar atrophy rates in terms of maintaining the ordering of the three structures.

**Table 4:** NnU-Net segmentation accuracy metrics based on dice coefficients for automated segmentation of BIOCARD and ADNI test datasets for amygdala, ERC, TEC, anterior hippocampus, and posterior hippocampus.

	Dice similarity coefficient	
	BIOCARD	ADNI
Amygdala	0.90 ± 0.025	0.87 ± 0.041
Entorhinal cortex	0.81 ± 0.051	0.83 ± 0.059
Trans-entorhinal cortex	0.81 ± 0.057	0.86 ± 0.067
Anterior hippocampus	0.96 ± 0.009	0.94 ± 0.023
Posterior hippocampus	0.96 ± 0.011	0.92 ± 0.038

### 3.3 BIOCARD and ADNI predict ordered atrophy: ERC/TEC ≈ Amygdala > Hippocampus

**Fig. 5:**
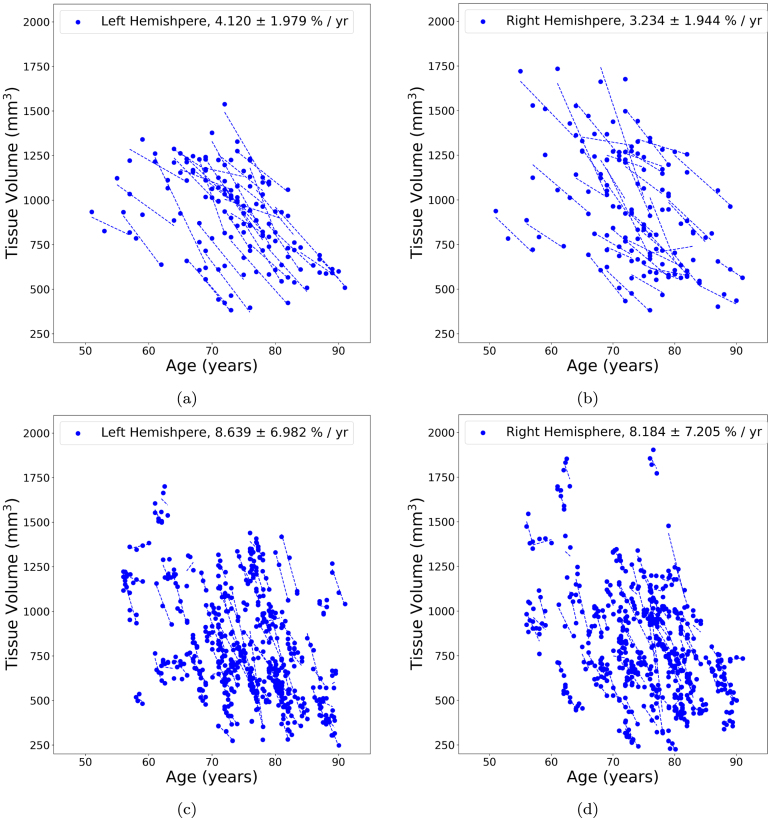
Volume atrophy rates in BIOCARD MCI/AD groups for the ERC/TEC left and right hemispheres ((a), (b)) and ADNI AD groups ((c), (d)). Dots represent volume at certain age; line fitted through all time points for each subject.

Longitudinal MRI-based volume trajectories revealed a consistent ordering of regional atrophy rates across disease stages in both the BIOCARD and ADNI datasets. Fig. 5 shows the trajectories of MCI/AD subjects, as reflected by the atrophy rates of each ERC/TEC region. The left and right columns correspond to the left and right hemispheres, respectively, and show ERC/TEC atrophy rates for BIOCARD MCI/AD subjects (top row) and the ADNI AD subjects (bottom row).

**Fig. 6:**
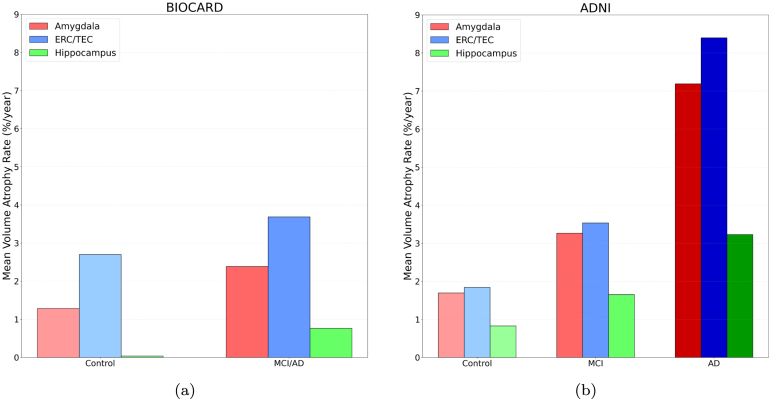
Volume atrophy rates in BIOCARD (a) and ADNI (b) for the amygdala (red), ERC/TEC (blue), and hippocampus (green). The average ages of subjects were BIOCARD Control 71.24 years, MCI/AD 73.36 years, ADNI Control, 75.26 years, MCI 73.73 years, AD 75.09 years.

Figs. 6 shows the summaries of the BIOCARD and ADNI cohorts in the MTL structures, exhibiting the stereotyped monotonic behavior of ERC/TEC ≈ Amygdala > Hippocampus in controls, MCI and AD groups. Specifically, the hippocampus atrophy rates in both datasets are markedly lower than the amygdala and ERC/TEC. In BIOCARD, the hippocampus atrophy rates are 0.04% and 0.76% in control and MCI groups, respectively. The amygdala atrophy increases dramatically in both groups to 1.29% and 2.38%, with the ERC/TEC increased as well to 2.70% and 3.69%. In ADNI, the hippocampus atrophy rates are 0.83%, 1.65% and 3.23% in control, MCI and AD, respectively, with the amygdala 1.69%, 3.26% and 7.19%, and the ERC/TEC 1.84%, 3.53% and 8.40%.

Subjects diagnosed with MCI or AD exhibit greatest atrophy in the entorhinal ERC/TEC with the highest rate of annualized volume loss, highlighting its role as an early and sensitive marker of AD-related neurodegeneration. Strikingly, the amygdala also demonstrated a remarkable early atrophy relative to that of the hippocampus in early disease. This ordering is preserved across left and right hemispheres, and across cohort studies, suggesting a stereotyped progression of degeneration.

**Fig. 7:**
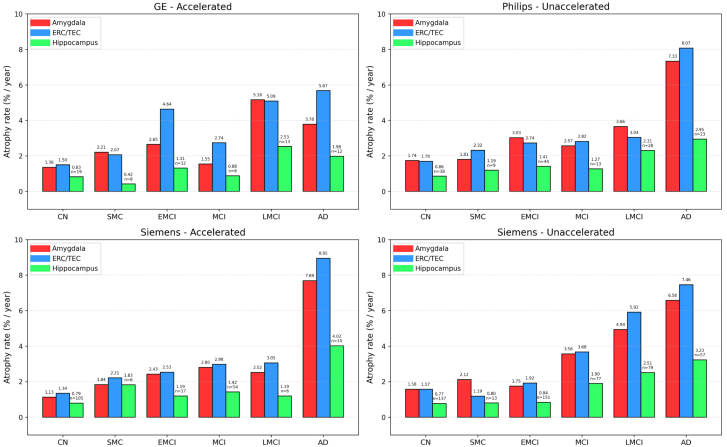
Volume atrophy rates stratified by diagnosis groups and scanner vendors (GE and Siemens, accelerated and unaccelerated acquisitions), demonstrating consistent trends across acquisition condition. n denotes the number of subjects in each disease group.

We examined the effect of scanner variation and T1-weighted acquisition protocols on estimated volume atrophy rates. Fig. 7 summarizes atrophy rates across the diagnostic groups for multiple acquisition protocols, including Accelerated Sagittal IR-FSPGR (GE), MPRAGE (Philips), and both standard and accelerated sagittal MPRAGE (Siemens). While absolute MTL volumes show significant variability across vendors, statistical analysis using the Kruskal–Wallis test with false discovery rate (FDR) correction reveals no significant differences in volume atrophy rates across vendors within each disease group. This robustness supports the use of atrophy rate as a reliable metric in multi-site and multi-scanner studies and enhances its potential for clinical translation where imaging protocols are often heterogeneous.

### 3.4 Amygdala and ERC/TEC measures improve early MCI classification beyond hippocampal atrophy

**Fig. 8:**
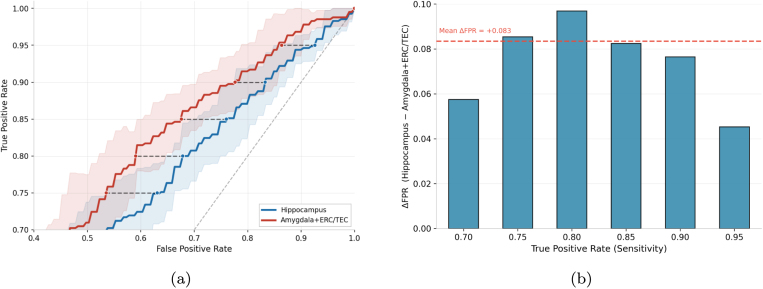
Comparison of hippocampus-only and amygdala+ERC/TEC models for CN versus EMCI classification. (a) Partial ROC curves showing classifier performance at high-sensitivity operating points. Shaded regions indicate the standard deviation across the 10 repeated stratified cross-validation runs. (b) Mean reduction in false-positive rate across sensitivity levels ranging from 0.70 to 0.95. Bars represent the mean ΔFPR across the repeated cross-validation runs, and the dashed red line denotes the overall mean reduction.

To assess the clinical value of amygdala and ERC/TEC measurements, we performed binary classification between CN and EMCI group and compared the performance with that of conventional hippocampal biomarkers. Fig. 8 summarizes the classification results. The classification performance was moderate, consistent with the subtle structural alterations during the earliest clinically detectable stages of AD. The combined amygdala + ERC/TEC model achieved a mean AUC of 0.665 ± 0.044, outperforming the hippocampus-only model, which achieved 0.635 ± 0.036. Statistical comparison with a Wilcoxon signed-rank test demonstrated that the improvement provided by the amygdala+ERC/TEC model was significant (p = 0.0059). Across sensitivity levels from 0.70 to 0.95, the average ΔFPR remained consistently positive, demonstrating that the amygdala+ERC/TEC model generally achieved lower FPRs than the hippocampus-only model while maintaining the same sensitivity. The average specificity increase is 8.3% across sensitivity levels ranging from 0.70 to 0.95, with gains of 9.7% and 8.2% at sensitivities of 0.80 and 0.85. These findings suggest that the improved classification performance of the combined amygdala+ERC/TEC model is primarily driven by increased specificity, reducing the number of cognitively normal individuals incorrectly classified as EMCI while maintaining comparable sensitivity. The significant improvement over the hippocampus-only model further indicates that amygdala and ERC/TEC volume and atrophy measures provide complementary diagnostic information beyond hippocampal atrophy and may enhance the detection of early AD-related structural changes.

### 3.5 ERC/TEC laminar thickness atrophy predicts volume atrophy

**Fig. 9:**
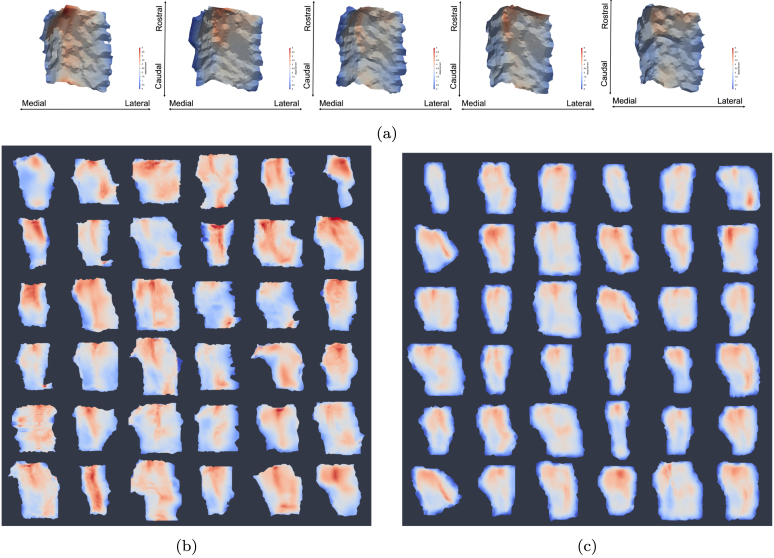
(a): Cortical thickness scalar field of ERC/TEC surfaces across five time points of one subject (scale bar showing thickness range of 1–5 mm). (b) and (c): Collage of thickness (on a scale of 1–5 mm) on inner surface of ERC/TEC from subsets of (b) ADNI and (c) BIOCARD.

Scalar field reconstructions of laminar ERC/TEC thickness are shown as follows. Fig. 9(a) shows the scalar field distribution of cortical thickness at five time points for a single individual in the ADNI cohort. The thickness color bar extends from 1 mm (blue-white) to 5 mm (red) from which the atrophy rates are calculated at each vertex. Figs. 9(b) and 9(c) are collages of surfaces from subsets of ADNI and BIOCARD. Cortical thickness is defined as the median of the vertex-wise thickness distribution derived from the scalar field.

**Fig. 10:**
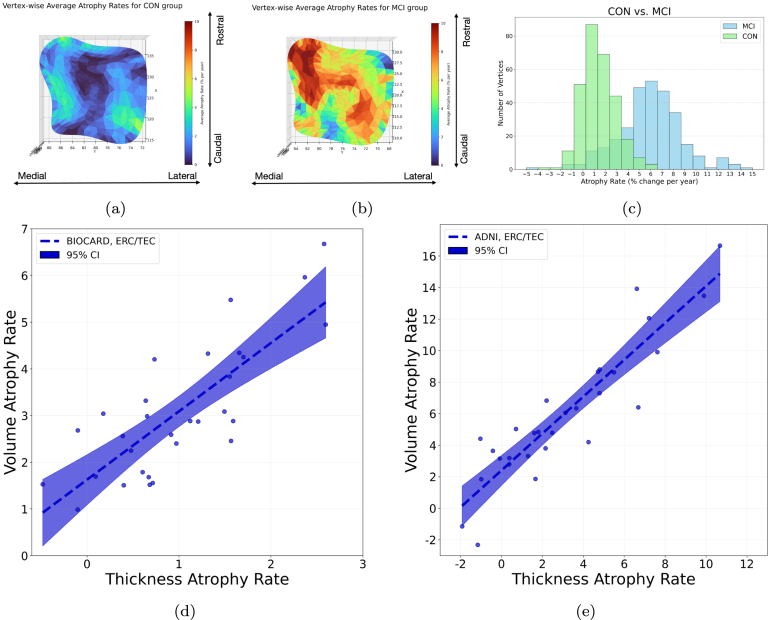
Averaged vertex-wise thickness atrophy rates, superimposed on baseline population target surface for (a) control and (b) MCI groups, with corresponding histogram analysis (c). Volume versus thickness atrophy rates in (d) BIOCARD and (e) ADNI.

Consistent with the volumetric trends, vertex-wise cortical thickness mappings of the ERC/TEC surface further reveal a graded spatial pattern of atrophy across clinical stages. Histogram analysis of vertex-wise annualized atrophy rates (Fig. 10(c)) shows that control subjects (Fig. 10(a)) exhibit lightly clustered, near-zero atrophy rate, whereas MCI subjects (Fig. 10(b)) display a heavy-tailed distribution with elevated atrophy, indicating more extensive and accelerated degeneration. This progression in both spatial extent and statistical distribution of atrophy further underscores the ERC/TEC region’s sensitivity to early AD-related neurodegeneration.

To explore the anatomical underpinning of volume atrophy in ERC/TEC, we examined the relationship between volume loss and cortical thinning. Figs. 10(d) and 10(e) depict volume atrophy rates (y-axis) versus cortical thickness atrophy rates (x-axis). Regression analysis indicates a linear correlation between ERC/TEC volume atrophy rate and cortical thickness loss, as derived from surface displacement measurements. In MCI/AD subsets of both datasets, the volumetric reductions are closely tied to laminar cortical surface thickness as manifest across the geometry of the surface. These results imply that ERC/TEC atrophy measured through volumetrics mirrors underlying laminar deterioration, affirming the biological validity of these imaging biomarkers. Such multi-dimensional confirmation strengthens the argument for ERC as a sentinel region in the early neurodegenerative cascade.

### 3.6 Subregional amygdalar atrophy coupled to high-field atlasing

**Fig. 11:**
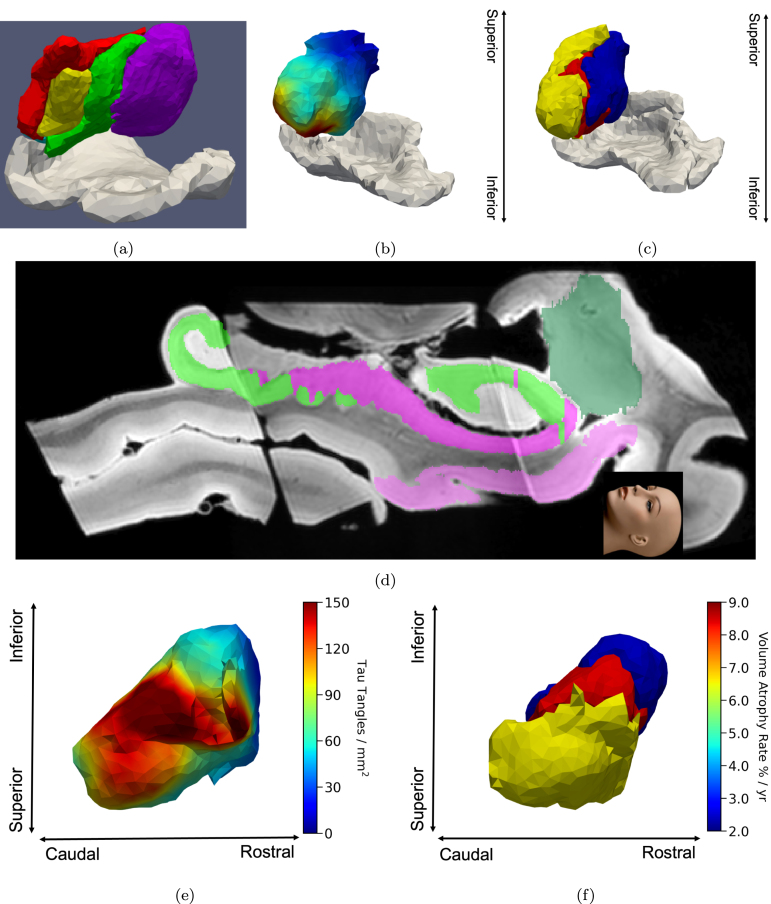
(a): Amygdala subregions including BMA (8.24%, yellow), BLA (8.31%, green), CMA (6.34%, red), and LA (2.52%, purple). (b) NFT densities of amygdala in an AD postmortem sample (Stouffer et al., 2023); outlines of ERC and TEC surfaces shown in white mesh. (c): Average volume atrophy rates of subregions in deformed high-field atlas in the MCI/AD group from ADNI. (d): A section through the high-field MRI depicting the caudal (left) to rostral extent (right) of the amygdala (dark green) and its position relative to the rostral extent of the entorhinal cortex (light pink). (e): Tau NFTs of the amygdala looking superiorly from the entorhinal cortex (avatar face looking up). (f): Same Volume atrophy rates as (c) viewed from the same angle as in (e).

To resolve the spatial specificity of atrophy within the amygdala, we conducted subregional analyses using atlas-based deformations, mapping high-field amygdala reconstructions into four major regions: BMA, BLA, CMA, and LA, as shown in Fig. 11(a). MRI volumes were then mapped onto these subregions, and atrophy rates were calculated for each. Volume loss was found to be notably heterogeneous across amygdala subnuclei. The BLA, BMA and CMA regions exhibited the greatest atrophy rates in MCI subjects, while lateral amygdala was comparatively spared. Interestingly, these findings mirror known patterns of tau pathology observed in histological studies and align with the amygdala’s structural and functional connectivity to other MTL regions, such as the ERC and hippocampus.

Figs. 11(a)–11(c) demonstrate that atrophy in the amygdala based on the high-field atlases is consistent with the actual tau profile as reconstructed by (Stouffer et al., 2023) using high-field digital pathology integration of the tau misfolded protein. Fig. 11(b) shows neurofibrillary tangle (NFT) densities of amygdala in the AD postmortem sample including BMA, BLA, CMA, and LA. The white mesh depicts the outlines of ERC and TEC surfaces. Fig. 11(c) shows the volume atrophy rates in the deformed high-field atlas for the BMA (8.24%), BLA (8.31%), CMA (6.34%), and LA (2.52%) subregions in a subgroup of MCI/AD from ADNI. Notice that the lowest atrophy occurs at the identical lateral side of the amygdala with lowest tau density profile.

### 3.7 MRI atrophy measures are correlated to rostral disease and are associated with tau burden as demonstrated by the high-field atlasing and tau PET imaging.

Fig. 11(d) illustrates a section of the rostral extent of the tauopathy and MRI atrophy within the epicenter of the high-field digital pathology of NFT tangles, as well as the localized tau density profile (Fig. 11(e)) and volume atrophy rates (Fig. 11(f)) measured via the MRI high-field atlas mapping. Consistently, the lowest decrease in the NFT density profile is at the identical lateral side as the decreased atrophy marker in LA.

**Fig. 12:**
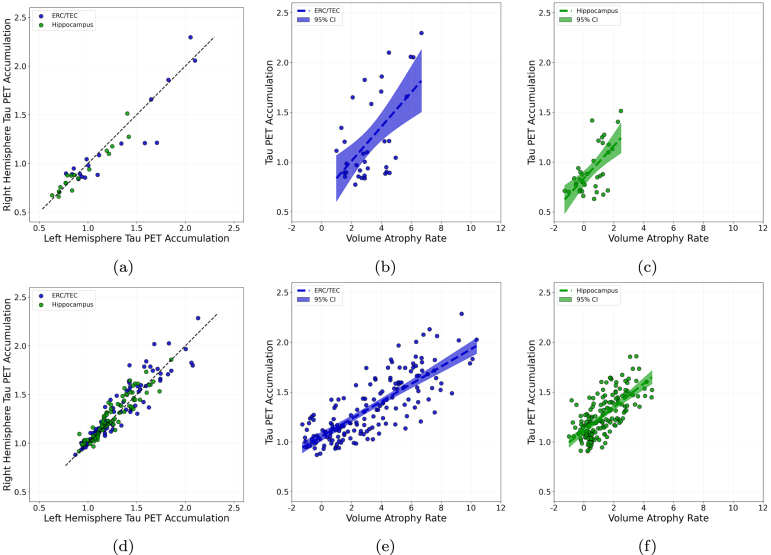
(a) and (d): Tau PET consistency of the ERC (blue) and hippocampus (green); left structures plotted on x-axis and right structures on y-axis; straight line is the y=x linear trend. (b), (c), (e), and (f): Linear regression of tau PET accumulation (y-axis) versus volume atrophy (x-axis) in the ERC ((b) & (e)) and hippocampus ((c) & (f)), with each group shown with shaded area denoting the 95% Confidence Interval (CI). Top and bottom rows respectively show the BIOCARD and ADNI cohorts for MCI/AD subjects.

Fig. 12 examines the relationship between the region-specific atrophy markers and tau burden as measured using PET scans. The left column shows tau markers measured in each hemisphere for MCI/AD groups in the BIOCARD (Fig. 12(a)) and ADNI (Fig. 12(d)) cohorts, where tau PET accumulation is plotted against the line y=x, demonstrating strong inter-hemispheric symmetry. The fact that green points for the hippocampus are predominantly in the lower left part of the y=x while the ERC/TEC in the upper right quadrant demonstrates the delayed atrophy progression of the disease in the hippocampus relative to the ERC/TEC. This bilateral consistency suggests that the PET-derived biomarkers are stable and robust across hemispheres, enhancing confidence in their use for individual subject-level tracking. The consistency also served as a further validation of our segmentation, volume quantification, and PET normalization procedures resulting from the alignment pipeline of tau images to the segmented MRI volume. The right two columns demonstrate the relationship between tau PET accumulation and volume atrophy within the ERC/TEC (middle) and hippocampus (right) in the MCI/AD subjects. Regression lines fitting through individual data points reveal consistent trends across regions, underscoring the fact that the atrophy marker is highly correlated with tau PET accumulation and is region specific. Elevated tau accumulation, as measured via PET imaging, strongly predict accelerated volume loss in corresponding anatomical regions.

## 4 Discussion

This paper explores quantifiable medial temporal lobe atrophy associated with specific regions implicated in Alzheimer’s disease. While the hippocampus has historically been emphasized in the study of Alzheimer’s disease-related neurodegeneration, our results demonstrate a consistent and reproducible ordering of atrophy (ERC/TEC ≈ amygdala > hippocampus) across two independent longitudinal cohorts, highlighting that these regions exhibit early and pronounced degeneration relative to the hippocampus. Given that the hippocampus is an elongated structure spanning nearly 4 centimeters, localized shape-based analysis offer greater sensitivity to localized atrophy than global volumetric measurements of the entire structure.

This paper has also examined the ERC/TEC as a laminar structure, establishing that ERC/TEC volume loss reflects underlying laminar cortical thinning through diffeomorphic surface reconstruction, providing a biologically grounded interpretation of MRI-derived atrophy. In addition, the use of high-field atlasing enables subregional characterization of amygdala degeneration, revealing preferential vulnerability in medial sub-nuclei, which aligns with known tau deposition patterns. Compared to standard volumetric biomarkers, the proposed framework offers a more nuanced characterization of disease progression better aligned with tau pathology, which has potential utility in identifying at-risk individuals before clinical symptom onset, stratifying patients for clinical trials, and tracking disease progression in response to therapeutic interventions.

Growing evidence suggests that the amygdala plays a substantially more important role in the early stages of AD than has traditionally been recognized. While classical AD imaging studies have focused primarily on the ERC and hippocampus, recent neuropathological, molecular imaging, and structural MRI studies indicate that the amygdala is affected early in the disease process and exhibits regionally selective vulnerability that may provide complementary information beyond conventional hippocampal biomarkers. Stouffer et al. (Stouffer et al., 2024) reviewed converging evidence from postmortem pathology, tau-PET imaging, connectivity studies, and structural MRI, demonstrating that the amygdala is an early site of neurofibrillary tangle accumulation and may participate in a medial temporal lobe pathway of tau propagation distinct from the classical entorhinal-hippocampal pathway. Multiple cross-sectional studies have highlighted the role of amygdala in progression to MCI and early AD dementia (Mori et al., 1997, Punzi et al., 2024, Mizuno et al., 2000, Goerlich et al., 2017), though the findings are not universal (Roh et al., 2011). Some studies have associated early amygdala atrophy with subjective cognitive decline progressing to MCI (Göschel et al., 2023) and with basolateral nuclear complex, anterior amygdala, and transitional area atrophy in cognitively normal individuals predicting cognitive decline within 2 years (Padulo et al., 2023). Others have highlighted early atrophy in more medial amygdala nuclei associated with amyloid and tau positivity in individuals who are cognitively unimpaired and in MCI (Salman et al., 2024, Yuan et al., 2026). These findings are similar to our study (Miller et al., 2015) demonstrating that AD-related amygdala degeneration is spatially selective rather than uniform across the structure. Significant atrophy in that study was localized primarily to the basomedial and basolateral nuclei, and high-dimensional shape markers were more sensitive than global volumetric measurements for distinguishing symptomatic AD subjects from controls, highlighting the early association of amygdala regional atrophy with cognitive decline. Similarly, Cavedo et al. (2011) used 3T MRI and radial atrophy mapping to characterize local amygdala structural alterations in AD. They reported 20%–30% tissue loss in the medial, central, and basolateral ventromedial nuclei, demonstrating that AD-associated degeneration preferentially affects specific amygdala subfields that are linked to hippocampal, olfactory, and cholinergic networks known to be disrupted in AD, providing a neuroanatomical basis for the observed regional vulnerability. Taken together, these studies support a revised view of the amygdala as an early and regionally selective target of AD pathology rather than a secondary structure affected only in later disease stages. The convergence of neuropathological, tau-PET, and high-resolution morphometric evidence indicates that specific amygdala subfields are involved during preclinical and prodromal AD and may provide clinically relevant information beyond traditional hippocampal biomarkers. Consistent with this framework, our study found that amygdala degeneration exceeded hippocampal degeneration across disease stages and that incorporating amygdala measures improved discrimination of early MCI beyond hippocampal atrophy alone.

Interestingly, while the amygdala plays a central role as an interconnected hub to critical brain regions in the memory circuit and is clearly an early actor exhibiting pathologic changes during neurodegeneration, relatively less is known about the molecular and cellular level changes in the amygdala in AD. The amygdala innervates many of the brain structures crucially affected by early tau pathology in AD, including the ERC, hippocampus, basal forebrain nuclei, and olfactory bulb (Braak et al., 2006, Braak and Del Tredici, 2011). Experimental evidence implicates the amygdala in fear processing, anxiety, aggression, stress, motivation, feeding behaviors, and depression (Rasia-Filho et al., 2000, Schaefer and Gray, 2007, Stouffer et al., 2024). While not central to the classic symptomatology of AD, these behaviors constitute many of the diagnostic criteria for mild behavioral impairment (MBI), which is emerging as an early clinical marker of AD (Creese and Ismail, 2022) and consequently a support for a role of the amygdala in early AD. Additionally, there is evidence that the amygdala plays a role in long term memory modulation, particularly memories associated with strong emotion, and in working memory and attention (Schaefer and Gray, 2007), as traditionally implicated in the AD spectrum.

The functional and connective differences in subregions of the amygdala provide an interesting window into the possible circuit-level changes affected by the spatially-distributed pathology in the amygdala in AD and related dementia. Padulo et al. (2023), for instance, showed that atrophy in specific amygdala subfields distinguishes cognitively normal individuals who later convert to MCI, highlighting the sensitivity of amygdala subnuclei analysis in preclinical disease. Salman et al. (2024) also reported that amygdala subnuclei volumes are associated with tau burden in preclinical AD, whereas global amygdala and hippocampal volumes exhibit reduced sensitivity at this stage and only become associated with tau pathology as disease progresses. Our studies here and others (Tsuchiya and Kosaka, 1990, Kromer Vogt et al., 1990, Stouffer et al., 2023) further demonstrated that tau pathology in the amygdala in AD is distributed in specific spatial patterns. Generally, studies of postmortem human tissue in AD agree that the more lateral regions in the amygdala have less tau pathology compared to the more medial regions (Tsuchiya and Kosaka, 1990, Kromer Vogt et al., 1990, Stouffer et al., 2023, Yushkevich et al., 2021), though the anatomic definitions of regions vary.

Hence, targeting of more granular regional differences in atrophy and in tau pathology accumulation fosters identification of both additional and more specific biomarkers for AD. Concurrently, examination of networks and additional pathophysiological processes offers the opportunity to link seemingly discordant biomarkers and ripen our identification and understanding of the AD disease process. For instance, Chen et al. (Chen et al., 2021) showed that structural atrophy, hypometabolism, and cognitive decline reflect complementary but partially independent processes, with network-level dysfunction (e.g. default mode network hypometabolism) contributing beyond structural loss alone. Examining the MTL at a circuit-level, the medial amygdala subregions are shown to be linked to the anterior hippocampus, ERC, and locus ceruleus (Stouffer et al., 2024), suggesting possible neuronal links for spread of pathologic tau in AD (Vogel et al., 2020) between each of these historically implicated areas. In future, improved understanding of the subregional and cell-type specific changes in the amygdala could provide insight into the potential presence of multiple circuits of tau spread through the medial temporal lobe and the circuit-level dysfunction associated with cognitive and neuropsychiatric symptoms in AD.

The proposed computational framework has several aspects of translational feasibility. The segmentation pipeline is based on nnU-Net, a self-configuring and widely adopted framework that has demonstrated strong generalization across datasets and requires minimal manual tuning. The processing pipeline has been implemented in a modular manner and is available via a dedicated GitHub repository to facilitate reproducibility and adoption. The LDDMM framework is also available through multiple diffeomorphic mapping frameworks, with the high-field amygdala atlas and ERC cortical surface mapping now available as well through the repository.

## CRediT authorship contribution statement

**Michael I. Miller:** Writing – review & editing, Writing – original draft, Supervision, Project administration, Methodology, Funding acquisition, Formal analysis, Conceptualization. **Yi Xie:** Writing – review & editing, Writing – original draft, Visualization, Validation, Software, Methodology, Investigation, Formal analysis, Data curation, Conceptualization. **Kaitlin M. Stouffer:** Writing – review & editing, Methodology, Formal analysis, Data curation, Conceptualization. **Can Ceritoglu:** Writing – review & editing, Software, Data curation. **Jiabei Li:** Data curation. **J. Tilak Ratnanather:** Writing – review & editing. **Laurent Younes:** Writing – review & editing, Methodology, Formal analysis, Conceptualization. **Arnold Bakker:** Writing – review & editing, Data curation. **Nisha Rani:** Data curation. **Marilyn S. Albert:** Writing – review & editing, Funding acquisition. **Juan C. Troncoso:** Writing – review & editing, Resources, Formal analysis, Data curation, Conceptualization. **Meaghan Morris:** Writing – review & editing, Resources, Formal analysis, Data curation, Conceptualization.

### Funding

This work was supported by the 10.13039/100000002National Institutes of Health (U19-AG033655, P30-AG066507, P41-EB031771, R01-EB020062, 1F30AG077736-01, T32GM136577), 10.13039/501100008982National Science Foundation (2309683) and the Kavli Neuroscience Discovery Institute.

## Declaration of competing interest

The authors declare the following financial interests/personal relationships which may be considered as potential competing interests: Michael I. Miller reports a relationship with AnatomyWorks that includes: equity or stocks. If there are other authors, they declare that they have no known competing financial interests or personal relationships that could have appeared to influence the work reported in this paper.

## Figures and Tables

**Fig. C.13:**
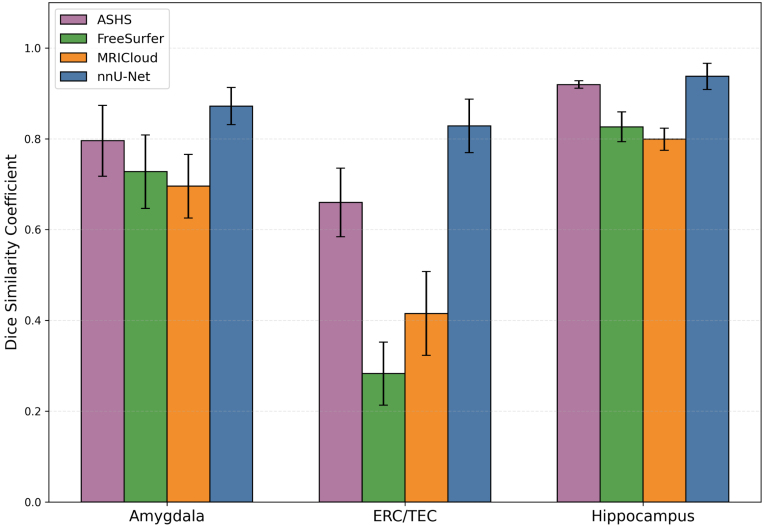
Dice coefficients for amygdala, ERC/TEC, and hippocampus segmentation on the test datasets, comparing ASHS, FreeSurfer, MRICloud, and nnU-Net. Error bars represent standard deviation.

**Fig. C.14:**
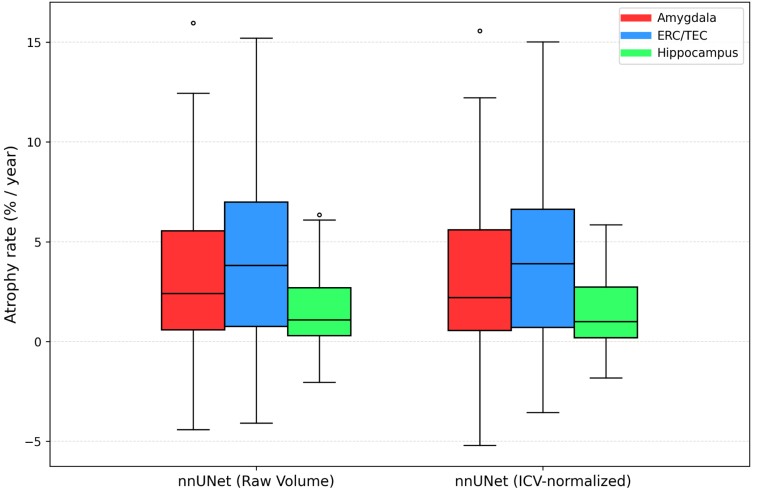
Comparison of volume atrophy estimates with (right) and without (left) intracranial volume (ICV) normalization in 177 MCI subjects, each with three or more 3T Siemens MPRAGE scans.

## Data Availability

The authors do not have permission to share data.

## Appendix A BIOCARD data security

BIOCARD study participants are assigned a study ID and a LetterCode at the time of consent. Study participants, datafiles, brain images and specimens are identified by a study ID only, with no personal identifiers included. All datafiles, as well as the MRI and PET scans, use these identifiers. These study-specific identifiers allow cross-reference of all collected data, including biofluids, PBMCs, iPSCs, brain tissue, and other derived samples.

The BIOCARD database houses all the datafiles in the study and is protected through multiple levels of password protection. Protection is provided by the central Johns Hopkins Enterprise system, using multifactor authentication protocols that protect access to the database itself. In addition, each individual in the BIOCARD study that accesses the database must have individually assigned permissions (implemented by the Data Management and Statistics Core). The brain images are stored in an XNAT database which is also protected by the central Johns Hopkins Enterprise system. Access to the images in the XNAT database requires a separate set of authorization, which is provided by the Neuroimaging Core.

Note that when data and images are shared with outside investigators, a unique study ID, known as the JHU Anonymous ID, is created for each study participant specifically. Additionally, the datafiles released are considered ‘Limited’ datafiles in that all data records are free of the personal identifiers specified in section 164.514(b)(2)(i) of the Federal Privacy rule, and indirect identifiers that could potentially compromise the anonymity of participants are suppressed (e.g., date of birth).

## Appendix B ADNI data security

The Alzheimer’s Disease Neuroimaging Initiative (ADNI) employs a centralized informatics framework through its Informatics Core at the Laboratory of Neuro Imaging (LONI). Before being made available through the Image and Data Archive (IDA), all data undergo a standardized processing workflow which includes subject identifier validation, potential identity information removal, quality control, and metadata extraction (Toga et al., 2024). Sensitive metadata and protected health information (HI) embedded in imaging files (e.g. Digital Imaging and Communications in Medicine (DICOM), Neuralimaging Informatics Technology Initiatives (NIFTI)) are modified or removed in accordance with limited dataset requirement proposed by Health Insurance Portability and Accountability Act (HIPAA) prior to upload, and additional safeguards are applied to more sensitive data types. Data Sharing and Publication Committee oversees data access to ADNI data by an approval process, with all user activity logged to ensure transparency and accountability. The infrastructure of ADNI dataset further incorporates secure data transmission via encryption, controlled access systems, and robust storage mechanisms to maintain data integrity and security.

## Appendix C MTL segmentation performance comparison

Fig. C.13 shows segmentation comparison between ASHS, FreeSurfer, MRICloud, and nnU-Net on the 30 held-out test set. nnUNet shows a high Dice metric for the ERC label while maintaining strong performance in amygdala and hippocampus. The relatively lower Dice similarity coefficients observed in FreeSurfer segmentations can be attributed to its reliance on the Desikan–Killiany atlas, which provides a gyral-based parcellation not specifically designed for the anatomical specificity of the medial temporal lobe structures.

The nnUNet demonstrated relatively high performance specifically because it was trained on segmentaiton labels specifically focussed on the medical temporal lobe structures, amygdala, ERC, and hippocampus. The acquired intensity, spatial and contextual feature variation specific to the MTL provide the network the specificity associated to its accurate delineation of the medial temporal lobe structures.

We further examined the effect of whole-brain atrophy on regional volume loss, comparing the atrophy rate using intracranial volume normalization. A subset of MCI subjects were chosen and normalized. Intracranial volume (ICV) was computed as the sum of gray matter, white matter, and cerebrospinal fluid volumes within the cranial vault. Each time point volume was normalized by the corresponding ICV with the normalized atrophy rate then computed. Fig. C.14 shows the box-plots of the volume atrophy rates. A minimal difference in mean atrophy rate was observed showing 4.18% for the ERC, with normalization decreasing to 4.05%.
